# Cochlear implantation under local anesthesia in 117 cases: patients’ subjective experience and outcomes

**DOI:** 10.1007/s00405-021-07061-4

**Published:** 2021-09-06

**Authors:** Aarno Dietz, Thomas Lenarz

**Affiliations:** 1 Department of Otorhinolaryngology, Kuopio University Hospital, PO. Box 100, 70029 Kuopio, Finland; 2 Department of Otorhinolaryngology, Hannover Medical School, Hannover, Germany

**Keywords:** Cochlear implantation, Local anesthesia, Consious sedation, Hearing preservation, Elderly

## Abstract

**Purpose:**

To report the outcomes and the patients’ subjective experience of cochlear implantation (CI) performed under local anesthesia (LA). To describe a new form of intraoperative cochlear monitoring based on the patients subjective sound perception during CI.

**Methods:**

In this retrospective case–cohort study, 117 patients underwent CI under LA with (*n *= 58) or without conscious sedation (*n *= 59). Included were primarily elderly patients with elevated risks for general anesthesia and recently patients with residual hearing eligible for electro-acoustic stimulation (EAS) (*n *= 27), in whom hearing could be monitored during the electrode insertion. A 500 Hz test tone was presented and the patient reported  of subjective changes in loudness, leading to a modification of the insertion. A questionnaire was sent to all patients in which they assessed their subjective experience.

**Results:**

All patients were successfully operated under LA without the need to intraoperatively convert to general anesthesia. 90% of the patients reported that the surgery was a positive experience. The vast majority, 90% of patients were satisfied with the overall treatment and with intraoperative pain management and 84% of the patients would opt for local anesthesia again. Cochlear monitoring by the patients’ subjective sound perception enabled for atraumatic insertions as all EAS patients could hear the test tone up to the end of the surgery.

**Conclusions:**

CI under LA was well tolerated and recommended by the vast majority of patients. In addition, it offers the possibility to monitor the patients’ hearing during the electrode insertion, which may help to prevent insertion trauma.

## Introduction

With increasing life expectancy more than 900 million people will have disabling hearing loss (HL) by the year 2050 [1, 2]. Untreated HL has a well-established association with anxiety, depression, cognitive decline, and loss of social activity and autonomy [2–6]. Adequate hearing rehabilitation, including cochlear implantation (CI), is essential to alleviate these serious consequences of HL. Due to ageing demographics, patients eligible for CI will become constantly older.

Currently, CI is a standard care for severe-to-profound HL and has been found to be effective also for elderly patients (≥ 65 years) [7–12]. Although CI surgery is considered to be relatively safe also in elderlies [13, 14], the presence of comorbidities may increase their risks for general anesthesia, which explains why many elderly candidates are unwilling to undergo operative treatment. Recently, postoperative cognitive dysfunction has been attracting  more attention [15, 16]. This refers to a delayed deterioration of cognitive function in elderly patients after general anesthesia, and is thought to be associated with the increased vulnerability of elderly people to the neurotoxicity of volatile and intravenous anesthetics [17, 18]. For these reasons, the avoidance of general anesthesia in elderly patients is highly desirable.

With respect to CI surgery, there is an increasing interest in hearing preservation and structure preservation, since more favorable hearing outcomes can be achieved when the integrity of the inner ear can be preserved. The preservation of residual acoustic hearing is especially beneficial, as this  may allow electric-acoustic stimulation (EAS), which often improves the quality of sound and the speech intelligibility in noise with the cochlear implant [19]. For patients with residual hearing, CI under local anesthesia (LA) would provide the possibility to monitor reliably their residual hearing in  the ear being operated  by presenting a test sound during electrode insertion. In this way, patients can give the surgeon immediate feedback whenever the sound changes or becomes attenuated during the insertion of the electrode. The insertion may then be adjusted and imminent insertion trauma possibly prevented.

To date, the literature about CI under LA is limited, mostly consisting of small sample reports [20–27]. The aim of this study is to report about the surgical and subjective outcomes of CI under LA and its special application in hearing preserving surgery.

## Materials and methods

This retrospective study complied with the Declaration of Helsinki on ethical principles for medical research involving human subjects and had institutional approvals.

One hundred and seventeen consecutive patients underwent CI under LA at the Kuopio University Hospital, Finland (KUH) and the Hannover Medical School, Germany (MHH) from 01.10.2014 to 31.12.2020. Data on the surgical results were collected from the medical files. Patients were given the option between surgery under general anesthesia or  under LA. Preoperatively, all patients were thoroughly counselled about the procedure and its different steps. More recently, we also offered the option of LA to patients with residual hearing and who were eligible for EAS. The acceptable preoperative hearing thresholds for EAS were defined as ≤ 60 dB at 250 and 500 kHz. Of the 117 patients, 79 patients were ≥ 65 years of age and 27 patients were EAS candidates.

The preoperative workup followed each institute’s routine protocol including high resolution computed tomography (HRCT) or cone beam computer tomography (CBCT) and magnetic resonance imaging (MRI) for surgical planning and the exclusion of inner ear malformations and/or retrocochlear pathology.

Surgical intervention under LA requires seamless cooperation and communication between the patients and the surgical team. Oral communication with CI recipients is most often challenging, especially in noisy surroundings, such as in the OR. Whenever feasible, a hearing aid was fitted with a long tube in the contralateral ear or connected with an audio link device for best possible communication. For deaf patients, with no speech intelligibility in their contralateral ear, we used a tablet computer with a large font size to convey written information. In cases, with concomitant visual problems, preoperatively we taught the patients straightforward  tactile communication skills.

**Fig. 1:**
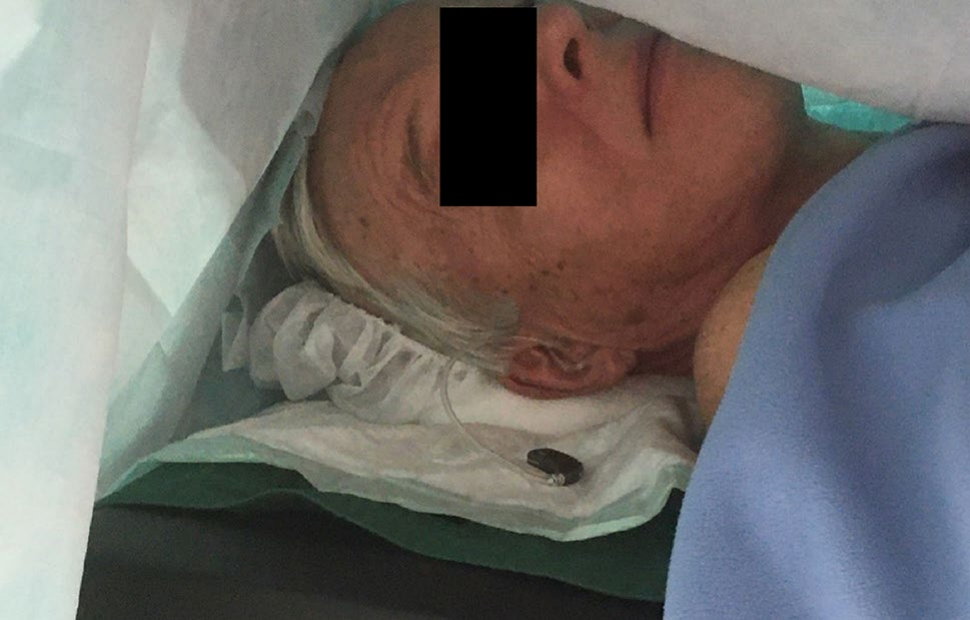
Operation room’s setup. Plenty of space under the draping for patient comfort and to facilitate communication via tablet computer. Note: the hearing aid is connected to a long sound tube to prevent feedback. The hearing aid can be also connected via an audio link to a microphone clip for even better communication

During the OR preparation, special attention was paid to positioning the patient comfortably on the OR table. The draping was arranged to allow plenty of space above the patient’s face to prevent claustrophobia and to facilitate communication (Fig. 1). The irrigation hosepipe of the otologic drill was wrapped around an infusion warmer to heat the irrigation fluid of the drill to prevent a caloric reaction and vertigo during mastoid drilling. Intraoperative monitoring included electrocardiogram, non-invasive measurements of blood pressure, heart rate and pulse-oximetry. In this context, it is noteworthy, that facial nerve monitoring cannot be used in CI under LA.

**Fig. 2:**
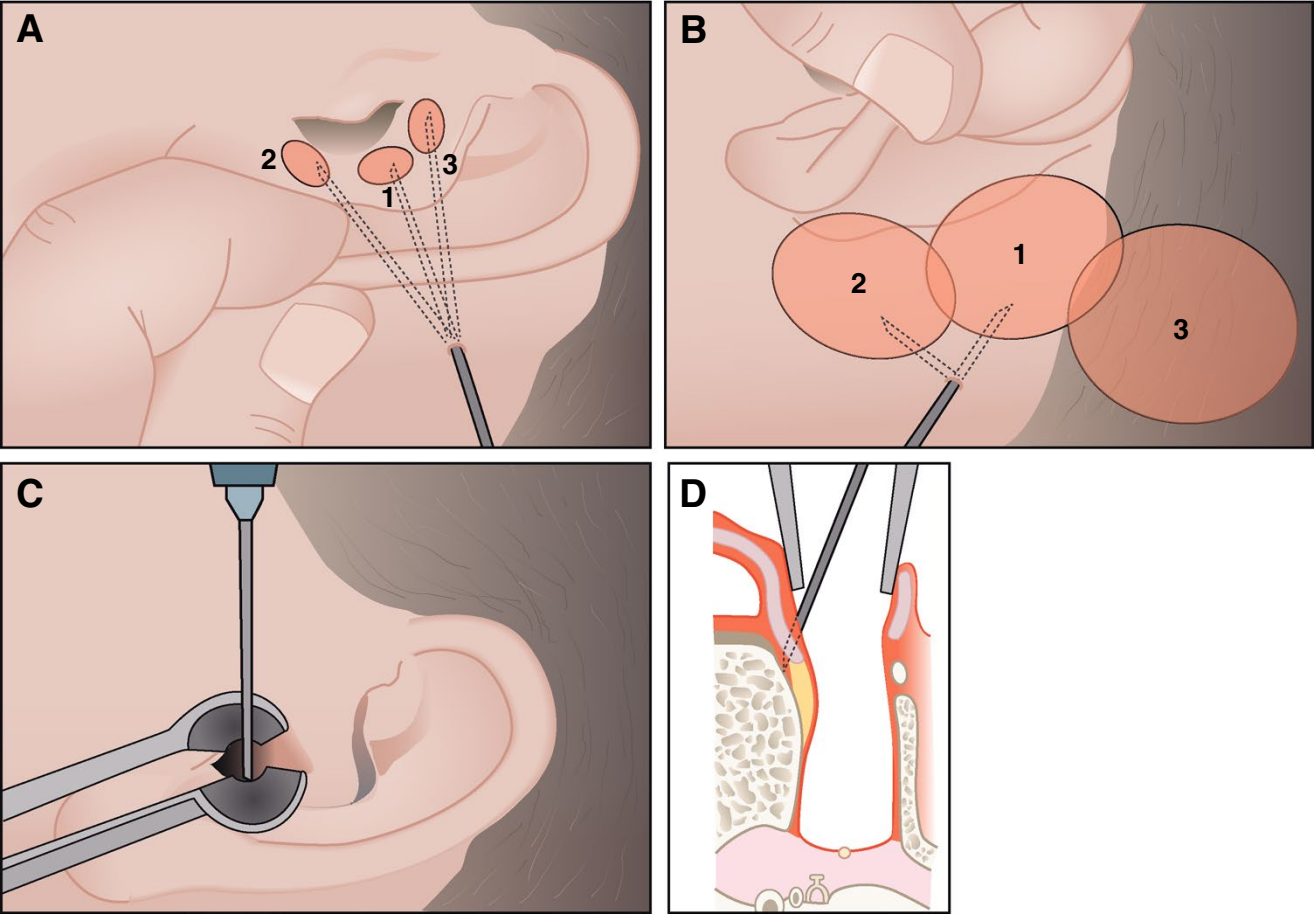
Illustration on the application of the local anesthetic. **A** 3–5 ml lidocaine is infiltrated from behind the ear under the conchal cartilage at the level of the outer ear canal. **B** Infiltration of 10–15 ml lidocaine in the retroauricular region (1 and 2) and at the planned site of the implant bed (3). **C**, **D**. Infiltration of the posterior bony ear canal. The needle should be placed through the cartilage under the periosteum of the posterior bony ear canal

At the KUH, an intra-venous single dose of fentanyl (2 µg/kg) was administered just before the start of each operation. At the MHH, promethazine 25 mg (intramuscular) and pethidine 50–100 mg (intramuscular) was used as premedication. In  the infiltration anesthesia of the retroauricular area and the external auditory meatus lidocaine 1% and epinephrine 1:50.000 (LcE) were used (Fig. 2). The usual amount of LcE varied  between 10 and 20 ml.

If the patient felt uncomfortable during the procedure, a light sedation was evoked  by an initial bolus of 10–20 mg propofol and a continuous infusion of propofol (1 mg/kg/h). In patients, who were preoperatively planned for additional conscious sedation (i.e., moderate sedation) received either a continued propofol infusion (1–3 mg/kg/h) or dexmedetomidine (0.6–1 µg/kg/h) infusion.

The surgical procedure was chosen according to the individual's anatomy. CI surgery was performed via a transmastoid-posterior tympanotomy or a modified suprameatal approach (described elsewhere [23]) with different electrodes from four manufactures. After opening the facial recess, pieces of gelfoam soaked with lidocaine were applied onto the promontorium. The round window membrane was protected from lidocaine with gelfoam saturated with dexamethasone (10 mg/ml).

In patients with residual hearing, their hearing was monitored intraoperatively during electrode insertion. A constant tone of 500 Hz was presented via an insert earphone and patients were instructed to report promptly about any changes in  the perceived tone. Whenever the perceived sound changed or became attenuated, the insertion was modified or stopped. In some cases, the electrode had to be retracted until the tone reverted to its original loudness and quality and then the insertion proceeded at  a different trajectory. For hearing preservation surgery, we used the concept of partial insertion previously described by Lenarz et al. [28]. The rationale for deliberately inserting an array only partially is that it provides the better hearing preservation results associated with  shorter electrodes but will also allow to forward the array deeper into the cochlear (via a follow-up procedure) in cases, if the residual hearing deteriorates. For exclusively electric stimulation, deeper insertions would provide better neural coverage with possibly better hearing performance. 

After wound closure, electrically evoked compound action potentials were recorded in the postoperative mode with custom-settings and the patients reported about the volume and pitch of the perceived stimuli. After surgery, patients were transferred  directly to the ENT ward. A cone beam computed tomography was obtained on the first postoperative day for the assessment of adequate electrode placement and possible trauma.

A questionnaire with overall six questions regarding the patients’ subjective assessment of treatment in general and their experience of surgery under LA was send to the patients (Table 1a). The questionnaire sent in the year 2020 and patients  completed and returned it anonymously.

**Table 1:** a, b The patient survey (a) The questionnaire (b) The results of the survey

a	
Q1	I am pleased with the treatment
Q2	My experience about the surgery was positive
Q3	The pain relief during surgery was adequate
Q4	I felt safe during the surgery
Q5	I felt good after the surgery
Q6	I would choose local anesthesia for surgery again

b					
	I strongly agree	I agree	I do not know	I disagree	I strongly disagree
Q1	65 (78%)	15 (18%)	1 (1%)	1 (1%)	1 (1%)
Q2	59 (71%)	16 (19%)	6 (7%)	2 (2%)	0 (0%)
Q3	67 (81%)	12 (14%)	2 (2%)	1 (1%)	1 (1%)
Q4	66 (80%)	13 (16%)	3 (3%)	1 (1%)	0 (0%)
Q5	58 (70%)	18 (22%)	2 (2%)	5 (6%)	0 (0%)

	Yes	No	I do not know
Q6	70 (84%)	11 (14%)	2 (2%)

## Results

### Surgery under local anesthesia

Patient characteristics, surgical data and the devices used are listed in Table 2. All patients were successfully operated under LA. There was no need to convert intraoperatively to general anesthesia in any patient. Surgery was well tolerated by nearly all patients. 58 patients received additional conscious sedation, whereas 59 patients were operated on with exclusively LA. In general, drilling the mastoidectomy was endured well, however, some patients, especially those with residual hearing, complained about the loud noise and vibration. Pain during drilling was experienced by some patients when approaching the middle fossa dura plate, thus we believe that  it is advisable to leave some air cells overlying the middle dura plate. We did not notice any adverse reactions such as dizziness or vertigo during the insertion of the electrode. However, most patients (also many with complete deafness) often reported acoustic sensations during insertion, including tinnitus and differently pitched sounds. Intraoperative impedance- and ECAP measurements led to a hearing sensation in all patients. It is noteworthy, that intraoperative test protocols were  avoided, since they may cause overstimulation and pain; therefore, individualized postoperative test protocols were applied. Nearly all patients were also able to differentiate different pitches according to the stimulated electrode contact along the cochlear partition. Unlike after procedures conducetd under general anesthesia, there was no need for surveillance in the recovery room. There were no major complication. One patient developed a perforation of the tympanic membrane after the suprameatal approach, which required myringoplasty at a later stage. Two patients experienced vertigo lasting for several days; however, the vestibular function was found intact.

**Table 2:** Patient characteristics, surgical data and hearing preservation

Total no. of patients: 117	No
Patients	
Kuopio University Hospital	82
Hannover Medical School	35
Age (at surgery)	
Range (yrs)	27–88
Mean (yrs)	67
Median (yrs)	71
Sex	
Male	68
Female	49
Device	
Advanced bionics	24
Cochlear	22
Med-El	60
Oticon	11
Side	
Right	65
Left	52
Surgical approach	
Transmastoid-posterior tympanotomy	101
Modified suprameatal	16
Conscious sedation	
Yes	58
No	59
Hearing preservation	
Patients eligible for EAS^a^	27
Intraoperative hearing preservation^b^	27
Partial insertion	25

^a^Preoperative threshold at 250 Hz and 500 Hz ≤ 60 dB (HL)

^b^Patients were able to perceive the presented test tone throughout the insertion process up to the end of the surgery

### Hearing preservation

There were 27 patients with residual hearing acceptable for EAS. In these patients, hearing monitoring with a constant sound stimuli was performed during the insertion of the electrode. All patients were able to clearly perceive the presented test tone and were able to perceive  changes such as fading and blurring during the insertion. The loudness of the test tone most often recovered when the electrode array was retracted or if the insertion trajectory was modified. In 25 patients, the electrode was only partially inserted to achieve the best possible hearing preservation. All patients reported that they were able to hear the test sound from the beginning of the insertion process up to the end of the surgery. On the first postoperative day, threshold testing revealed that there was successful hearing preservation in all of these patients.

### Patients’ subjective assessment

The majority i.e., 83 out of 117 patients (response rate 71%), returned the questionnaire and 97% of the patients were satisfied with the overall treatment received. With respect to the LA, 90% stated that the overall experience was positive and 95% reported, that the intraoperative pain relief was sufficient. Almost all, 96% of the patients, expressed that they felt safe during the whole course of surgery and 92% of the patients reported feeling well postoperatively. Finally, 70 out of 83 patients (84%), would opt for CI under LA were it needed, only eleven (14%) were not willing to undergo such a procedure under local anesthesia again.  Two patients could not decide (Table 1b).

## Discussion

 Due to the ageing of the population, the number of elderly individuals developing severe-to-profound HL will increase rapidly. For this reason, CI under LA is of increasing clinical relevance, since potential CI patients are becoming older, with most of them having significant comorbidities, which make them poor candidates for general anesthesia. There is an overall need to improve the provision of CI therapy especially in the elderly population. It has been reported that the oldest patients (81–100 years) have fewer possibilities to be offered even an evaluation for CI. The researchers found that, for unknown reasons, health care professionals often appear to avoid discussing CI treatment with older patients. [29] However, old patients themselves may not actively consider CI treatment because of their fear of the invasive procedure and its possible complications. Indeed, we have  observed this mindset in many of our oldest patients. We found that being able to offer them  the option to undergo surgery under LA had helped many patients to decide in favor of CI. Therefore, the dissemination of this method may help to increase the utilization of CI in the elderly population.

Currently, the literature about CI under LA is still sparse and represents  mostly rather small patient samples. This present report is based on our experience of CI surgery under LA in over one hundred patients and thus represents the largest patient cohort reported to date. Our main finding is that surgery under LA is feasible and surprisingly well tolerated in the overwhelming majority of patients. Over 90% of the patients were satisfied with their treatment; they reported that the intraoperative pain management was adequate and they felt safe during the procedure. Similarly, 84% of the patients would choose CI under LA again, if faced with this decision, i.e., for the contralateral ear. The most common complaint was the loud sound and vibrations during drilling, which was expressed especially by patients with residual hearing. Our results are in accordance with the study of Pateron et al. in which the patient’s experience has also been systematically enquired [22]. It is noteworthy  that there has been a long tradition for conducting otologic procedures under LA, especially in middle ear surgery [30]. The safety of middle ear procedures under LA has been well demonstrated in earlier studies [30–32]. Some publications stated that patients have reported that they  feel safer when surgery is performed under  LA, which is in agreement with our findings. [31, 32] In our study, 97% of patients felt safe during surgery, which is surprising considering the fact that especially elderly patients were preoperatively rather anxious about the surgery. In contrast to our report, all other studies investigating CI under LA routinely applied  conscious sedation. In our patient cohort, conscious sedation was used in about every second patient. In fact, with increasing experience, the necessity for additional conscious sedation became less frequent, and it is currently reserved only for those patients who start to feel uncomfortable during the course of surgery. Pain management was considered adequate by the majority patients. This confirms that the infiltration anesthesia provided mostly satisfactory pain control; this was achieved  by adding  Gelfoam soaked with lidocaine to critical structures, such as the round window niche and the middle dura plate.

In comparison to previous studies, we are first to report about CI under LA without conscious anesthesia. The relatively large number of those patients in our cohort (*n *= 59) documents its feasibility. This has important clinical implications with regard to OR resources and costs; an anesthesiologist is not required to be present in the OR, and patients can be directly transferred to the ward without the compulsory stay in the recovery unit. With increasing experience of CI under LA, we found no additional benefit from conscious sedation. On the contrary, during conscious sedation patients often fall asleep and startle when waking up, which bears a high risk for surgical complications, especially during drilling or electrode insertion. In addition, older patients can be easily disoriented by sedation, which significantly impairs their cooperation and communication with the OR team.

We found that good cooperation and communication with the patient during the surgery is of paramount importance, which is why conscious sedation may be in fact counterproductive. Therefore, we currently use only very light sedation or preferably no sedation at all, to keep patients co-operative and addressable throughout the surgery. This is especially important in patients undergoing hearing preservation surgery in whom hearing is monitoring intraoperatively during the insertion. With conscious sedation, patients would not be sufficiently attentive to be able to report about changes in the presenting sound during insertion.

The application of CI under LA yields novel and interesting opportunities for hearing preservation surgery, since it makes possible real-time hearing monitoring during electrode insertion. This approach carries the potential for a more atraumatic insertion with possibly better postoperative hearing preservation. The opportunity to test the patient's hearing during the insertion of the electrode might well represent the most reliable method to avoid trauma during the insertion procedure. Accordingly, our data show, that all patients were able to hear the stimulating sound up to the end of the surgery. Therefore, it is reasonable to assume that the insertion itself induced no severe mechanical trauma to the inner ear. However, we cannot make any conclusions about the long-term hearing preservation results, since the follow-up data are in the collection phase. It is of clinical relevance on how the residual hearing evolves in this special group of patients, in whom intraoperative hearing preservation had been reliably documented.

An additional current aspect for adopting CI under LA would be to treat patients with impaired pulmonary function, e.g., following COVID-19 disease, in whom general anesthesia is not recommended. At present, there is a growing number of patients who have lost their hearing after  COVID-19 infection due to labyrinthitis; these individuals would be a specific but illustrative reason for CI under LA. 

It is obvious that CI under LA cannot be applied in every patient. Patients must be sufficiently motivated to undergo an invasive procedure without general anesthesia. The anticipated surgical time should not exceed 1.5 hours, and therefore, the application of this technique is usually restricted to individuals with a normal mastoid and cochlear anatomy. In addition, it may be contraindicated in patients with critical ischemic heart disease in whom the blood pressure levels must be kept within narrow margins, which may be easier to achieve with invasive hemodynamic monitoring under general anesthesia.

The strength of the present study is the large patient sample and the systematic enquiry of the patients’ subjective experience. The response rate was adequate to allow us to draw reliable conclusions. However, questionnaire-based studies may be subject to a recall bias, i.e., errors in the accuracy of the patient’s recollection. To control for the overall response bias, the questionnaires were administered anonymously. The main limitation of this study relates to its retrospective nature. Prospective studies will be  needed to investigate the advantages and limitations of CI under LA and should address safety, patient-related issues, cost-effectiveness as well as  POCD. With regard to hearing preservation surgery, a longer follow-up will be needed to assess, whether the subjective hearing monitoring enabled by operating on awake patients will indeed exert  a positive effect on the hearing preservation results. Prospective studies will be also needed to compare hearing preservation results in patients undergoing CI under LA combined with  hearing monitoring against those obtained with surgery under general anesthesia and electrocochleography monitoring.

## Conclusion

CI surgery under LA is generally well tolerated and represents a viable alternative for elderly patients with comorbidities and elevated risks for general anesthesia. It may help to extend  the provision of CI to more elderly patients, since advanced age and most chronic conditions will  no longer represent a contraindication for CI surgery. In addition, CI under LA may also be a promising approach for hearing preservation surgery as it allows for real-time monitoring of the patient’s hearing during electrode insertion, which may help to reduce insertion trauma.
